# Expert consensus recommendations to improve diagnosis of ATTR amyloidosis with polyneuropathy

**DOI:** 10.1007/s00415-019-09688-0

**Published:** 2020-01-06

**Authors:** David Adams, Yukio Ando, João Melo Beirão, Teresa Coelho, Morie A. Gertz, Julian D. Gillmore, Philip N. Hawkins, Isabelle Lousada, Ole B. Suhr, Giampaolo Merlini

**Affiliations:** 1 Department of Neurology, French National Reference Centre for Familial Amyloidotic Polyneuropathy, CHU Bicêtre, Université Paris-Saclay APHP, INSERM U1195, 94276 Le Kremlin-Bicêtre, France; 2 Department of Neurology, Graduate School of Medical Sciences, Kumamoto, Japan; 3 Ophthalmology Service, Hospital de Santo António, Porto, Portugal; 4 Centro Hospitalar Do Porto, Porto, Portugal; 5 Mayo Clinic, Rochester, MN USA; 6 National Amyloidosis Centre, University College London, London, UK; 7 Amyloidosis Research Consortium, Boston, MA USA; 8 Department of Public Health and Clinical Medicine, Umeå University, Umeå, Sweden; 9 Amyloidosis Center Foundation, IRCCS Policlinico San Matteo, San Matteo, Italy; 10 Department of Molecular Medicine, University of Pavia, Pavia, Italy

**Keywords:** ATTR amyloidosis, ATTRv, Diagnosis, hATTR, Peripheral neuropathy, Transthyretin amyloidosis

## Abstract

Amyloid transthyretin (ATTR) amyloidosis with polyneuropathy (PN) is a progressive, debilitating, systemic disease wherein transthyretin protein misfolds to form amyloid, which is deposited in the endoneurium. ATTR amyloidosis with PN is the most serious hereditary polyneuropathy of adult onset. It arises from a hereditary mutation in the *TTR* gene and may involve the heart as well as other organs. It is critical to identify and diagnose the disease earlier because treatments are available to help slow the progression of neuropathy. Early diagnosis is complicated, however, because presentation may vary and family history is not always known. Symptoms may be mistakenly attributed to other diseases such as chronic inflammatory demyelinating polyradiculoneuropathy (CIDP), idiopathic axonal polyneuropathy, lumbar spinal stenosis, and, more rarely, diabetic neuropathy and AL amyloidosis. In endemic countries (e.g., Portugal, Japan, Sweden, Brazil), ATTR amyloidosis with PN should be suspected in any patient who has length-dependent small-fiber PN with autonomic dysfunction and a family history of ATTR amyloidosis, unexplained weight loss, heart rhythm disorders, vitreous opacities, or renal abnormalities. In nonendemic countries, the disease may present as idiopathic rapidly progressive sensory motor axonal neuropathy or atypical CIDP with any of the above symptoms or with bilateral carpal tunnel syndrome, gait disorders, or cardiac hypertrophy. Diagnosis should include DNA testing, biopsy, and amyloid typing. Patients should be followed up every 6–12 months, depending on the severity of the disease and response to therapy. This review outlines detailed recommendations to improve the diagnosis of ATTR amyloidosis with PN.

## Introduction

**Fig. 1:**
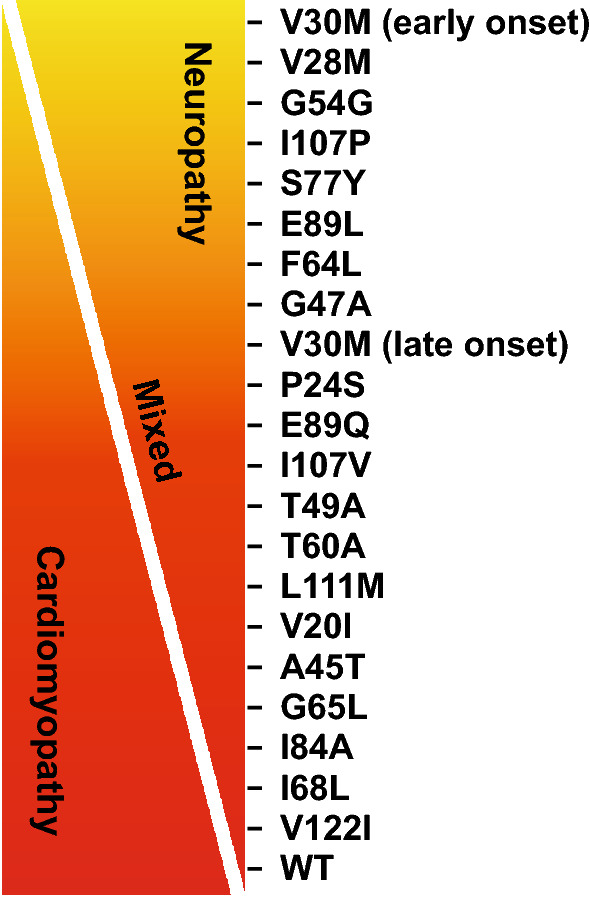
Genotype–phenotype correlations in ATTR amyloidosis. *ATTR* amyloid transthyretin, *WT* wild type. Reprinted with permission from Castano et al. [6]

Hereditary amyloid transthyretin (ATTRv; v for “variant”) amyloidosis with polyneuropathy (PN) is a rare multisystemic disease with predominant involvement of the peripheral nervous system and amyloid deposits in the endoneurium [1]. It was first described in endemic areas in Portugal and later in Japan and Sweden and is now considered a worldwide disease [2]. ATTRv amyloidosis has an autosomal-dominant mode of transmission because of a point mutation of the *TTR* gene [3]. Certain *TTR* mutations are associated predominantly with endoneurial amyloid deposition that results in polyneuropathy (most commonly *Val30Met*); others are associated with predominant cardiomyopathy or a mixed phenotype [4–6] (Fig. 1).

ATTRv amyloidosis is the most serious hereditary polyneuropathy of adult onset and a progressive, devastating, and life-threatening disease. Diagnostic delay varies in nonendemic regions from 3 to 4 years. Average survival from disease onset varies from 6 to 12 years, and cardiac involvement is often the cause of death [7, 8].

The disease is caused by abnormal transthyretin (TTR) protein that misfolds and aggregates to form amyloid fibrils that deposit in organs and tissue. It has long been considered an endemic disease with a high prevalence (~ 1/1000 persons). Early diagnosis is typically facilitated by positive family history, stereotypical neurologic manifestations such as length-dependent polyneuropathy and autonomic dysfunction [9], and presence of the unique TTR variant *Val30Met*. Gradually, it has been reported in many countries outside endemic areas with a sporadic presentation and is now well accepted as a globally prevalent disease. The estimated prevalence of ATTRv amyloidosis with PN worldwide is 10,000 (1/1,000,000 persons) [2].

## Purpose and methodology

The diagnosis of this rare disease is a challenge for the neurologist and is most often delayed by 3–4 years, which impacts patients’ functional and vital prognosis. Diagnostic delays occur for multiple reasons, but oftentimes misleading diagnoses are made because of sporadic, late-onset, highly varied clinical presentation patterns of various TTR variants [10]. In this review, we describe the main phenotypes of neuropathies of this disease and present simple tools to quickly confirm the diagnosis and to perform the minimal investigations needed to clarify the systemic extension of the disease.

Consensus recommendations for the suspicion and diagnosis of all forms of ATTR amyloidosis were developed through a series of development and review cycles by an international working group consisting of key amyloidosis specialists in collaboration with companies conducting research in ATTR amyloidosis (GSK, Ionis, Pfizer, Alnylam) and the Amyloidosis Research Consortium. These consensus recommendations were developed based on the published literature and the medical expertise of the international working group through in-person meetings along with refinement of the draft by telephone or email. The literature was surveyed using PubMed Central, and references were selected by the expert working group according to the relevance of the data. Recently, specific consensus recommendations were provided for cardiology ATTR amyloidosis [11]. This review describes the specific consensus recommendations for best practices in ATTR with predominant PN. It is intended to provide clinicians with an overview of important aspects of ATTR diagnosis that may facilitate rapid and accurate identification of the disease.

## Clinical presentation and suspicion index

### Clinical manifestations and phenotypes

#### Historical phenotypes in endemic areas

In endemic areas (such as Portugal, Japan, Sweden, Brazil), the main phenotype represents the hallmark of ATTRv with PN—a length-dependent small-fiber PN with dysautonomia—with manifestations mimicking those of diabetic neuropathy [12]. In these areas, the disease may not be as difficult to diagnose because it is aided by positive family history, high penetrance, and typical clinical presentation and by genetic counseling for, detection in, and follow-up of carriers of the mutant *TTR* gene [13]. Penetrance, however, is highly variable. For instance, in Portugal, the median age at onset is around 30 years [7], and 80% of mutation carriers are reported to exhibit the disease by age 50, whereas this number is only 11% in Sweden [14, 15].

Initial symptoms of ATTRv with PN vary but can include sensory symptoms such as pain, paresthesia, and numbness in the feet; autonomic dysfunction such as digestive disorders and erectile dysfunction; and general items such as fatigue, weight loss, and plantar ulcers [7, 16]. Sensory loss progresses with advancing disease and eventually extends to the lower limbs and to the hands and arms. More advanced disease may also involve loss of reflexes, reduced motor skills, and muscle weakness [4, 17].

#### Two clinical neuropathic phenotypes in late versus early onset in Val30Met variant

ATTRv amyloidosis is classified on the basis of age at onset, and symptoms before the age of 50 distinguish early from late onset [18]. Clinical presentation and disease course differ considerably between patients with early-onset and those with late-onset ATTRv with PN associated with the *Val30Met* mutation [7, 8, 18–21] (Table 1). Early-onset disease follows the classical course. In patients with early onset, penetrance is high (0.8) [14] and the disease is nearly always associated with a positive family history, initial symptoms of somatic or autonomic peripheral neuropathy, less severe disease course, and longer survival [19]. Late-onset disease tends to occur sporadically and typically presents with peripheral (not autonomic) neuropathy. In families with late-onset disease, there is a male predominance and low penetrance. At 50 years, sensorimotor symptoms begin in the lower extremities with disturbance of both superficial and deep sensation (mixed sensory loss) and relatively mild autonomic symptoms [19, 22]. In the Swedish population, amyloid fibril composition determines the phenotype; ATTR consisting of full-length TTR is associated with early onset and neuropathy, whereas a mixture of TTR fragments is associated with late onset, neuropathy, and cardiomyopathy [23].

**Table 1:** Characteristics of *Val30Met* early- and late-onset ATTR amyloidosis at the time of diagnosis and clinical course

	Early-onset *Val30Met* [7, 19]	Late-onset *Val30Met* [8, 19, 20]
Age at onset, years	< 50	≥ 50
Country	Portugal, Japan^a^, Brazil, Sweden^b^	Sweden^b^, France, UK, Italy, Japan, USA
Positive family history, %	94	48
Peripheral neuropathy, %	57	81
Autonomic neuropathy, %	48	10
Weight loss, %	5	0
Disease course		
Mean delay in need for aid in walking, years	> 5.6	3
Mean delay for wheelchair bound, years	10	6
Cardiac events	Progressive conduction disorders	Restrictive cardiomyopathy Cardiac insufficiency Progressive conduction disorders
Median survival, years	11	7.3
Cause of death	Cachexia Infection	Cardiac insufficiency Sudden death Cachexia or secondary infection

Reprinted with permission from Adams [18]

*ATTR* amyloid transthyretin

^a^Endemic areas, Nagano and Arao Kumamoto, Japan

^b^Patients with early- and late-onset disease are found in the endemic area in Sweden; all are believed to have a common Swedish founder [21]

#### Other clinical phenotypes in nonendemic countries

In nonendemic regions, four ATTRv amyloidosis phenotypes are reported [13, 24]. Small-fiber PN is not predominant and may occur in about 33% of patients. Also reported are length-dependent, all-fiber PN with diffuse areflexia and mixed sensory loss for pain, temperature, and proprioception [19] mimicking demyelinating polyneuropathy [25, 26]; multifocal neuropathy with onset in the upper limbs [8, 27]; ataxic neuropathy [24]; and exceptional motor neuropathy [26, 28, 29].

### Misdiagnosis

For people in nonendemic areas, diagnosis is likely to be missed. In these areas, 52–77% of cases occur with no family history of the disease [13, 24, 28], and presentation is variable. It has been reported that ATTRv with PN is suspected in only 26–38% of initial evaluations in these areas [24, 28]. Multiple misdiagnoses before the correct diagnosis of amyloid neuropathy have been reported in 20–40% of cases [25, 27].

Misdiagnoses depend on the initial clinical presentation of neuropathy (symptoms and signs). Common misdiagnoses (Table 2) [13, 25–27, 29–32] of patients before the correct diagnosis of ATTRv with PN include chronic inflammatory demyelinating polyradiculoneuropathy (CIDP), idiopathic axonal polyneuropathy, lumbar spinal stenosis, and, more rarely, diabetic neuropathy and AL amyloidosis. Increased awareness of this serious disease and its symptoms—as well as better knowledge of simple diagnostic tools, especially among neurologists—is essential to enable early diagnosis and optimal treatment of ATTRv with PN.

**Table 2:** Main misdiagnosis and red flags

Misdiagnosis	Incidence, %	Misleading features	Red flags	References
CIDP	13–15	SM 4 limbs Diffuse areflexia Albuminocytologic dissociation Demyelination on biopsy Demyelinating NCS	Pain Sensory loss (wrists) Autonomic dysfunction Upper limb weakness NCS	[26] [25] [30] [31]
Chronic axonal idiopathic PN	24–33	Axonal neuropathy in the elderly, seemingly idiopathic	Severity, disability, rapid Difficulties in walking	[13] [30] [27]
CTS	11	Paresthesia in the hands	No relief after surgery	[27]
Lumbar spinal stenosis	7.3	Progressive difficulty walking in the elderly Spinal stenosis on lumbar CT or MRI	Abnormal NCS Worsening in spite of surgery	[25]
Motor neuron disease Motor neuropathy, ALS	< 1	Upper limb and tongue amyotrophy Dysarthria Hand weakness	Abnormal sensory SNAP (NCS) No symptoms of upper motor neuron involvement	[32] [29]
Miscellaneous				
Alcoholic PNP		Small-fiber length-dependent PN	Alcoholism	[25]
Diabetic PNP		Small-fiber length-dependent PN Autonomic dysfunction	Rapid severity/duration of diabetes Difficulties in walking	[30]
Paraneoplastic neuropathy		Non-length-dependent sensory loss + ataxia Weight loss	No anti-onconeuronal antibody Negative findings on whole-body PET	[27]

*ALS* amyotrophic lateral sclerosis, *CIDP* chronic inflammatory demyelinating polyneuropathy, *CT* computed tomography, *CTS* carpal tunnel syndrome, *MRI* magnetic resonance imaging, *NCS* nerve conduction study, *PET* positron emission tomography, *PN* polyneuropathy, *PNP* peripheral neuropathy, *SM* sensorimotor, *SNAP* sensory nerve action potential

The disease course for late onset is more aggressive and has a shorter survival time than for early onset [18]. Initial symptoms of late-onset disease may also include sensory problems in upper limbs (33%) and walking disorders (11%) [24], and autonomic neuropathy may occur later in the disease in approximately 47–78% of these patients [24, 28, 30].

Amyloid can also be deposited in the heart, eyes, and leptomeninges, resulting in associated organ dysfunction and clinical symptoms. Cardiac involvement is usually asymptomatic at diagnosis but has been detected in up to 72% of patients when using cardiac imaging [30]. Cardiac hypertrophy (septal thickness > 12 mm) is found at presentation in 33 of 60 (55%) patients with late-onset *Val30Met*, predominantly in males [20].

Physicians should be aware of the leptomeningeal forms of ATTRv amyloidosis, which are associated with cerebral hemorrhage [33–36] and CNS dysfunction, typically with symptoms related to CNS impairment such as dementia, ataxia, spasticity, seizures, and stroke-like episodes [37–40]. The abnormal TTR protein deposited in the leptomeninges may be produced in the choroid plexus, making liver transplantation less effective in these patients [37].

### Suspicion index

**Fig. 2:**
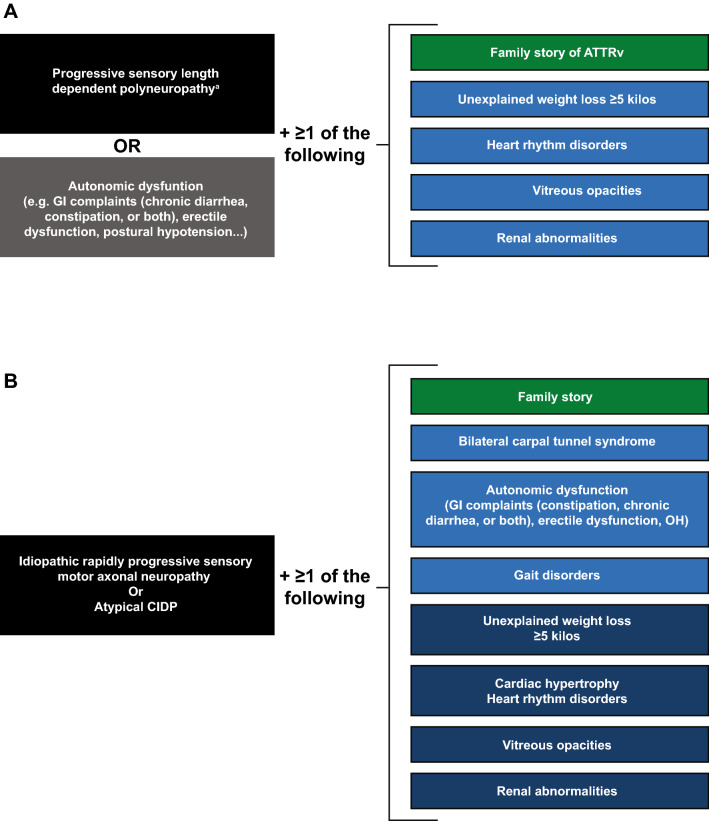
Suspicion index for diagnosis of ATTRv amyloidosis with PN. **a** In endemic areas. **b** In nonendemic areas. *ATTRv* hereditary transthyretin amyloid amyloidosis, *CIDP* chronic inflammatory demyelinating polyneuropathy, *GI* gastrointestinal, *OH* orthostatic hypotension. ^a^No diabetes, no alcohol abuse, vitamin B_12_ deficiency. Adapted with permission from Conceicao et al. [41]

Suspicion of ATTRv amyloidosis should be high for patients with progressive and disabling polyneuropathy, particularly in elderly patients. The disease should also be considered in patients with neuropathy plus at least one red flag symptom suggestive of multisystemic involvement (Fig. 2) [41].

For patients with a known family history of ATTRv amyloidosis, any onset of length-dependent axonal polyneuropathy predominantly affecting temperature and pain sensation, autonomic dysfunction, or cardiac arrhythmia signals a need to assess organ involvement.

For patients without a family history of amyloidosis, diagnosis of ATTRv amyloidosis should be considered if they have progressive idiopathic, axonal polyneuropathy, or atypical CIDP. Particular attention should be given to those who have autonomic dysfunction, early gait disorders, gastrointestinal disturbances and weight loss, carpal tunnel syndrome or previous surgery for bilateral carpal tunnel, concurrent cardiac abnormalities, or unexplained weight loss.

## Diagnosis

**Fig. 3:**
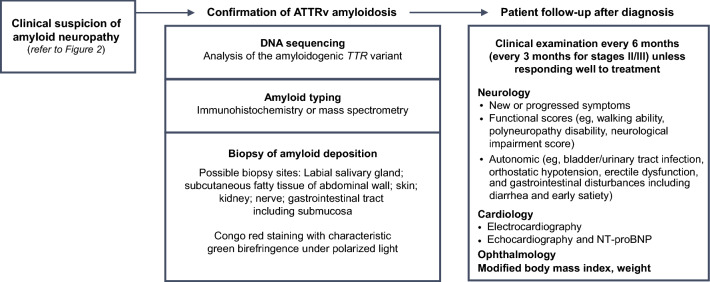
Diagnostic approach and patient follow-up. *ATTRv* hereditary transthyretin amyloid, *NT-proBNP* N-terminal fragment of the probrain natriuretic peptide, *TTR* transthyretin

Physicians should be aware of the clinical presentation and diagnostic approaches for patients with ATTRv amyloidosis with PN [10, 18, 25, 42–45] (Figs. 2, 3, Table 3). Clinical manifestations are diverse and nonspecific and may include neuropathic pain, loss of balance, carpal tunnel syndrome, and unexpected weight loss.

**Table 3:** Diagnostic tools for ATTR-PN

	*TTR* gene analysis	Amyloid detection	
		Biopsy^a^	DPD, PYP, HMDP scintigraphy
Advantages	Looking for 1 of the 130 amyloidogenic variants [10] Possible to rule out ATTRv if gene analysis is negative for a variant Fastest method to confirm ATTRv in case of neuropathy	Formal proof of amyloidosis in carriers of *TTR* variants and sporadic amyloid neuropathy	Noninvasive demonstration of cardiac amyloid with bone scintigraphy [43, 44]
Rules	*TTR* gene sequencing of the 4 exons	Congo red staining Examination under polarized microscopy Many sections often needed to detect a single deposit	
Limits	13 nonamyloidogenic variants, including Gly6Ser and Thr119Met [10] Possible delay of genetic results	Sensitivity for amyloid detection 60–80% [25] Dependent on experience and expertise of pathologist May be invasive and risky (cardiac) Time-consuming Several biopsy sites sometimes needed to find a deposit^a^	Radiolabeling if no light chain Sensitivity < 100% LV wall thickness > 12 mm in combination with abnormal heart/whole-body retention Heart/whole-body > 7.5 associated with the highest event rate [45]
Remarks	Some correlation between mutation and predominant organ involvement (e.g., heart, brain, eye)	False negative Amyloid light chain must be excluded	Complementarity of *TTR* gene analysis May avoid cardiac biopsy

*ATTR-PN* amyloid transthyretin polyneuropathy, *DPD* diphosphono-1,2-propanodicarboxylic acid, *HMDP* hydroxymethylene diphosphonate, *LV* left ventricular, *PYP* pyrophosphate

^a^At least one tissue biopsy should be performed to identify amyloid deposits and, if negative, another biopsy, preferentially mini-invasive (skin, labial salivary gland, abdominal fat), should be performed

### Diagnostic tools

There are only two main categories of diagnostic tools in ATTR-PN: *TTR* gene sequencing looking for *TTR* gene amyloidogenic variants and tools for detection of amyloid deposits including classical biopsy and, more recently, bone scintigraphy with diphosphono-1,2-propanodicarboxylic acid (DPD), hydroxymethylene diphosphonate (HMDP), or pyrophosphate (PYP) (Table 3). Staining a tissue biopsy (salivary gland, abdominal fat, or nerve tissue), typing for amyloid, and screening for *TTR* mutations by *TTR* gene sequencing are important measures for identifying amyloid neuropathy in sporadic cases presenting with rapidly idiopathic progressive axonal polyneuropathy of undetermined origin or atypical CIDP [13].

## *TTR* gene sequencing

The *TTR* gene, located in chromosome 18, is small (4 exons). More than 130 mutations can occur, most of which are pathogenic and amyloidogenic and are associated with varied phenotypes including predominant neuropathy, cardiomyopathy, and, more rarely, ocular and cerebromeningeal. A registry has been established to record the significance of mutations and phenotypes in ATTRv amyloidosis [10]. A few nonamyloidogenic variants have also been identified, including the polymorphism Gly6Ser; the discovery of such a variant has no significant value in a patient with idiopathic sporadic peripheral neuropathy. A TTR variant alone cannot confirm a diagnosis of ATTRv amyloidosis because of incomplete penetrance in carriers. Nevertheless, DNA sequencing of the *TTR* gene can be a useful approach in patients with idiopathic neuropathy to support or exclude a diagnosis of ATTRv amyloidosis and for predictive genetic counseling testing in healthy but potentially at-risk persons with a family history of ATTRv amyloidosis.

## Amyloid confirmation

### Biopsy

Staining of biopsy samples with Congo red and visualization of apple-green birefringence of Congo red-stained preparations under polarized light are crucial to confirm the diagnosis of disease and are indicative of the presence of amyloid fibrils. Finding amyloid deposits can be challenging, however, and negative biopsy results should not exclude a diagnosis [46, 47]. Mini-invasive biopsy include labial salivary gland biopsy, skin biopsy, and abdominal fat biopsy, which are preferred to invasive biopsies such as nerve biopsy and cardiac biopsy. The sensitivity of a biopsy can be impeded by inadequacies of the tissue samples; much depends on the site of the biopsy and on whether the biopsy includes nerve tissue (Table 4). In France and Portugal [48], biopsy of the salivary gland is preferred over abdominal fat aspiration, which is used in the USA, the UK, and other European countries except Sweden, where fat pad biopsy is used. The diagnostic sensitivity of a 3-mm-diameter skin punch biopsy at the distal leg 10 cm proximal to the lateral malleolus and proximal thigh is 70% [49, 50]. The minimal number of tissues to be examined for amyloid detection, including by mini-invasive biopsy, is two.

**Table 4:** Histologic and mass spectrometry methods for diagnosis of ATTR amyloidosis

Investigation	Sensitivity	Specificity	Aim
Biopsy site			
Sural nerve	79–80% TTR	High	Detecting amyloid deposits [24, 28, 30]
Labial salivary gland^a^	91% *Val30Met* early onset	High	Detecting amyloid deposits [77]
Abdominal fat pad^b^	14–83%	High	Detecting amyloid deposits [78]
Heart	~ 100%	~ 100%	Detecting amyloid deposits
Renal	92–100%	High	Detecting amyloid deposits [79–82]
Skin biopsy	70%	100%	Detecting amyloid deposits [49, 50]
Pathology test [83]			
Congo red staining	Medium–high	High	Detecting amyloid deposits
Polarized microscopy examination	High	High	Green birefringence
IHC with anti-TTR antibodies	High	Medium–high	–
Immuno-EM with anti-TTR antibodies	High	High	Detecting and typing amyloid fibrils
Mass spectrometry tests [84]			
LMD/MS	~ 100%	High	Determining specific type of amyloid deposits

Adapted with permission from Adams et al. [85]

*ATTR* amyloid transthyretin, *EM* electron microscopy, *IHC* immunohistochemistry, *LMD/MS* laser microdissection mass spectrometry-based proteomic analysis, *TTR* transthyretin

^a^Portugal and France

^b^USA, UK, the Netherlands, Germany, Sweden

#### Bone scintigraphy

Myocardial radiotracer uptake in bone scintigraphy with ^99m^technetium (Tc)-labeled 3,3-DPD, ^99m^Tc-labeled PYP, or ^99m^Tc-labeled HMDP could be useful in patients with peripheral neuropathy, amyloidogenic *TTR* mutation, and hypertrophic cardiopathy who have negative biopsy findings, and it may obviate the need for endomyocardial biopsy [44].

## Assessment of the extent of the disease

### Grading and staging other manifestations

Because ATTRv amyloidosis is a systemic disease, physicians should be aware of manifestations other than those of the peripheral nervous system, such as cardiac, ocular, and renal manifestations. A multidisciplinary approach is required to assess whether, through effects of autonomic dysfunction or amyloid deposition, other organs and systems are likely to be affected [18, 41].

#### Autonomic dysfunction

Autonomic dysfunction occurs in approximately 73% of patients with ATTR amyloidosis with PN and affects the gut, bladder sphincter, genital nerves, and cardiovascular system. Common symptoms include impotence (73% of male patients), gastrointestinal (GI) disturbance (53%), urinary incontinence (50%), and orthostatic dysregulation (46%) [51]. The most common symptoms seen in the GI system include weight loss (approximately 30% of patients), early satiety, and alternating constipation and diarrhea [52]. The incidence of GI disturbances increases as the disease progresses [53], and the onset of diarrhea earlier in the course of the disease is associated with shorter survival [54, 55].

#### Other organ involvement

Amyloid can also be deposited in the heart, eyes, kidneys, and, rarely, the leptomeninges, resulting in associated organ dysfunction and clinical symptoms. Cardiac involvement is usually asymptomatic at diagnosis but has been detected in up to 72% of patients through cardiac imaging [30] or cardiac multimodal imaging [56]. Cardiac involvement is associated with progressive myocardial infiltration, denervation, and conduction and rhythm disturbances. Systematic assessment and management of cardiac involvement is critical because cardiac manifestations worsen with disease progression and are more likely to cause death [18]. Ophthalmic manifestations have been reported in 20% (glaucoma and/or vitreous opacities) to 70% (dry eye) of patients with ATTR amyloidosis with PN [57–60].

#### Evaluation of the spread of the disease

Assessment of the spread of the disease is crucial for the detection of accompanying organ damage and requires a multidisciplinary approach by a neurologist (polyneuropathy, autonomic neuropathy), a cardiologist, an ophthalmologist, and a nephrologist or general health practitioner (Table 5) [61–71]. This is essential because the involvement of most organs, other than the nervous system, is latent but may have potentially major consequences—heart blocks, restrictive cardiomyopathy, glaucoma, renal insufficiency—for patients. Required evaluations for neuropathy include neuropathy impairment score (NIS), search for orthostatic hypotension, sudoscan, heart rate variability tests, Compound Autonomic Dysfunction Test for autonomic dysfunction, and Rasch-built Overall Disability Scale (RODS). Required evaluations for cardiac involvement include New York Heart Association (NYHA) score, electrocardiography (ECG), multimodal cardiac imaging [echocardiography (ECHO), magnetic resonance imaging (MRI), DPD, metaiodobenzylguanidine], complete ophthalmologic examination, and modified body mass index (mBMI). Biomarkers are also required for heart [N-terminal fragment of the probrain natriuretic peptide (NT-proBNP) and cardiac troponins] and renal [estimated glomerular filtration rate (eGFR), proteinuria] dysfunction (Table 5).

**Table 5:** Evaluation of disease progression at initial screening and follow-up

	Evaluation	Purpose	References
Neurologic manifestations			
A. Sensory motor neuropathy	Questionnaire		[61]
	Paresthesia, neurogenic pain	Small fiber loss	
	Gait disability	Large fiber loss	
	NIS (0–244)		
	Weakness in LL and UL	Large fiber loss	
	Sensory loss in toes and fingers	Small and large fiber loss	
	Tendon reflex loss in the four limbs	Large fiber loss	
	Examination		
	Pain and thermal sensory loss in the extremities in LL and UL (extension)	Small fiber loss	
Disability	Modified Norris test	Sensorimotor neuropathy	[62]
	FAP-RODS RODS	Overall disability Overall disability	[63] [86]
Locomotion	PND score	Autonomy to walk	
B. Autonomic neuropathy	CADT* (24-0)	Overall dysfunction	[62]
	COMPASS 31		[64]
	Sudoscan	Denervated sweat glands of the soles and palms	
	Orthostatic hypotension		[87]
	MIBG scintigraphy	Sympathetic cardiac denervation	
	Heart rate variability tests	Sympathetic and parasympathetic	
Non-neurologic manifestations			
C. Cardiac	ECG, Holter-ECG Cardiac staging	Looking for conduction block or arrhythmia	
	ECHO (strain)	Cardiac involvement	
	Cardiac MRI	Cardiac involvement	
	DPD, PYP, and HMDP scintigraphy	Cardiac amyloidosis	
	NT-proBNP	Cardiomyocyte stress	
	Cardiac troponin	Cardiomyocyte death	
	NYHA class	Extent of heart failure	
	NYHA class	Stage the extent of cardiac damage	[65]
D. Ocular	Slit-lamp examination Intraocular pressure Schirmer test Visual acuity	Vitreous opacities Ocular hypertension Dry eye (sicca syndrome)	
E. Kidney	Proteinuria eGFR	Renal dysfunction Renal insufficiency	
F. General condition	Weight mBMI	Nutritional status Nutritional status	
Quality of life	Norfolk QOL-DN	Disease-specific changes in QOL	[66]
	SF-36 QOL	Non-disease-specific changes in QOL	[67]
Overall scale for ATTR disease	Kumamoto neurologic scale	Sensory disturbances, motor weakness, autonomic dysfunction, and visceral organ impairment	[68, 69]
Sensory motor deficit in the limbs and autonomic dysfunction	NIS + 7, mNIS + 7	Composite score for clinical trial only	[70] [71]

*CADT* Compound Autonomic Dysfunction Test, *COMPASS* Composite Autonomic Symptom Score, *DN* diabetic neuropathy, *DPD* diphosphono-1,2-propanodicarboxylic acid, *ECG* electrocardiography, *ECHO* echocardiography, *eGFR* estimated glomerular filtration rate, *FAP-RODS* Familial Amyloid Polyneuropathy-Specific Rasch-built Overall Disability Scale, *HMDP* hydroxymethylene diphosphonate, *LL* lower limb, *mBMI* modified body mass index, *MIBG* metaiodobenzylguanidine, *MRI* magnetic resonance imaging, *mNIS* modified Neuropathy Impairment Score, *NIS* Neuropathy Impairment Score, *NT-proBNP* N-terminal fragment of the probrain natriuretic peptide, *NYHA* New York Heart Association, *PND* polyneuropathy disability, *PYP* pyrophosphate, *QOL* quality of life, *SF-36* 36-Item Short Form Survey, *UL* upper limb

#### Grading of the disease

Grading of the disease in each organ system involved is important for the follow-up of these patients. Grading allows detection of eventual disease progression and of organ complications that will require specific management (Tables 3, 5). The frequency of examinations should be determined by the severity and the systemic nature of the disease in each patient.

#### Follow-up

Patients with confirmed diagnoses should be routinely followed up to monitor for disease progression [18] (Fig. 3). Assessments should evaluate somatic neuropathy with locomotion (polyneuropathy disability score), severity of sensory motor neuropathy (NIS), autonomic dysfunction, manifestations with cardiac insufficiency (NYHA), biomarkers (ECG, ECHO, NT-proBNP), mBMI, renal dysfunction with eGFR, and proteinuria (Table 5). Assessments should be scheduled every 6–12 months, and that schedule should be maintained, depending on investigations.

The quantification of dysfunction caused by ATTRv amyloidosis depends on an array of clinical tests, including those that measure nerve conduction, autonomic neuropathy, manual grip strength, and lower limb function (Tables 5, 6) [7, 8, 18, 51, 72]. However, many of these tests have been used only in relatively small studies; further refinement and validation of these tests in larger patient cohorts are needed.

**Table 6:** Staging of ATTRv amyloidosis with PN, scales, and tools at baseline

Locomotion stage description [7]	Duration of stage, years		PND score [88]
	Early-onset Val30Met [7]	Late-onset Val30Met Other variants [8, 72]	
Stage 1 Disease limited to the lower limbs Walking without help Slight weakness of the extensors of the big toes	5.6 ± 2.8	2–4	PND I Sensory disturbances in extremities Preserved walking capacity PND II Difficulty walking but no need for a walking stick
Stage 2 Progression of motor signs in lower limbs with steppage and distal amyotrophies; muscles of the hands becoming wasted and weak Patient obviously disabled but can still move around with help	4.8 ± 3.6	2–3	PND IIIa 1 stick or 1 crutch required for walking
			PND IIIb 2 sticks or 2 crutches required for walking
Stage 3 Patient confined to a wheelchair or a bed, with generalized weakness and areflexia	2.3 ± 3.1	1–2	PND IV Patient confined to a wheelchair or a bed

Reprinted with permission from Adams [18]

*ATTRv* hereditary transthyretin amyloidosis, *PND* polyneuropathy disability

#### Consequences of diagnosis with ATTRv with PN

For patients with diagnoses of ATTRv with PN, early disease-modifying therapy may be beneficial [73, 74] and underscores the need for diagnosis as soon as possible. Genetic counseling is recommended for family members of patients, and therapeutic patient education is recommended for siblings and children [75, 76].

## Conclusions

Identification of ATTRv amyloidosis with PN can be challenging, particularly in nonendemic regions, and a high level of suspicion is required to diagnose patients as early as possible. Patients can present with heterogeneous symptoms and variable levels of disease severity, which often leads to a misdiagnosis of diabetic neuropathy or CIDP. Early and accurate diagnosis may also be confounded by a lack of family history and the presence of various phenotypes common to multiple disease conditions such as GI disorders. Older patient age at disease onset can also contribute to misdiagnosis because symptoms of ATTR amyloidosis may be confused with declines in systemic neurologic function that typically occur with normal aging.

In sporadic and potentially misdiagnosed cases, important tools for identifying amyloid neuropathy include *TTR* gene sequencing for amyloidogenic mutations, tissue biopsy (salivary gland, skin, abdominal fat, or nerve tissue) with staining, and amyloid typing. Because ATTRv with PN is a systemic disease, a holistic assessment approach should be used that includes consultation across multiple specialties (e.g., neurologists, cardiologists, ophthalmologists and eventually gastroenterologists, and nephrologists). Early and accurate diagnosis of ATTR amyloidosis allows early treatment and will potentially modify disease progression in patients.
